# Metagenomic Next-Generation Sequencing Can Clinch Diagnosis of Non-Tuberculous Mycobacterial Infections: A Case Report

**DOI:** 10.3389/fmed.2021.679755

**Published:** 2021-07-26

**Authors:** He Zhu, Min Zhu, Jia-Hui Lei, Ya-Li Xiao, Li-Min Zhao

**Affiliations:** ^1^Department of Respiratory and Critical Care Medicine, People's Hospital of Zhengzhou University, Henan Provincial People's Hospital, Zhengzhou, China; ^2^Department of Respiratory and Critical Care Medicine, Henan Provincial People's Hospital, Zhengzhou, China

**Keywords:** non-tuberculous mycobacteria, metagenomic next-generation sequencing, *mycobacterium avium complex*, diagnosis, case report

## Abstract

Non-tuberculou *Mycobacteria* (NTM) is ubiquitous in the environment and is conditional pathogen. Due to NTM and *Mycobacterium tuberculosis* belong to the genus *Mycobacterium*, their pathogenic mechanisms and clinical manifestations are similar. Therefore, NTM can cause tuberculosis-like lesions and lead to misdiagnosis. Early diagnosis and treatment greatly improve prognosis. However, traditional pathogenic microorganism detection has limitations, and it is difficult to accurately identify strains in clinical practice. Here, we report a 65-year-old man with NTM who presented with recurrent fever and cough. Computed tomography of the chest revealed a lung infection. The previous improper diagnosis and treatment did not improve his condition. With the aid of metagenomic next-generation sequencing, the pathogen was identified as *Mycobacterium avium complex*. Subsequently, he received accurate treatment and made significant improvements in clinical and radiology.

## Introduction

Non-tuberculous *Mycobacteria* (NTM) refers to a general term for a large group of mycobacteria other than *Mycobacterium tuberculosis complex* and *Mycobacterium leprae*. According to statistics, more than 190 species of NTM strains and 14 subspecies have been discovered so far, of which only a few are pathogenic to humans and belong to conditional pathogens (1, 2). Among pathogenic bacteria, the *Mycobacterium avium complex* (*M. avium complex*, MAC) is the mycobacterium with the most new species or subspecies (3). NTM disease means that the human body is infected with NTM and causes the disease of related tissues and organs, most commonly in the lungs. In recent years, as the infection rate of NTM has increased year by year, NTM pulmonary disease (NTM-PD) has become a common clinical disease, accounting for about 70–80% in the United States, while China currently has no specific information data in this area (4, 5). Because the clinical characteristics of the disease are similar to those of tuberculosis, it often leads to misdiagnosis and missed diagnosis, which delays treatment and seriously threatens human life and health.

NTM-PD often occurs in patients with original underlying diseases, especially chronic respiratory diseases, such as chronic obstructive pulmonary disease, bronchiectasis, and cystic pulmonary fibrosis. Due to the weakened autoimmune function, NTM infection cannot be effectively controlled, and eventually it progresses to NTM-PD (6, 7). The main clinical symptoms of NTM-PD are fever, cough and sputum. Due to the atypical clinical manifestations, it often needs to be differentiated from other diseases of the respiratory system. In the case of sterile species identification results, it can be misdiagnosed as *Mycobacterium tuberculosis* infection for a long time. Here, we report a case in which a patient's alveolar lavage fluid sample was identified by metagenomic next-generation sequencing (mNGS) and the final diagnosis was MAC lung disease.

## Case Presentation

We report a 65-year-old man who attended teaching hospital at Henan with a 10-day history of fever, intermittent cough, expectoration and wheezing. Five days prior to admission, the patient had been treated with anti-infective drugs at his local hospital (such as rifampicin, ethambutol, clarithromycin, piperacillin sodium and tazobactam sodium, and levofloxacin), but the therapeutic effect was not good. He had a past medical history of chronic obstructive pulmonary disease (COPD). He lived in the rural Henan province, China, and has no recent travel history out of the province.

Physical examination showed that his underarm temperature was 38 °C, pulse was 108 beats/min, breathing was 25 beats/min, and the patient had a barrel-shaped chest, partial collapse of the upper left anterior thorax, clear voice during percussion of both lungs, regular breathing, and scattered dry and wet rales on auscultation of the lungs.

The laboratory studies revealed elevated erythrocyte sedimentation rate (69 mm/h), C-reactive protein (61.77 mg/L), rheumatoid factor quantification (74.7I U/mL), procalcitonin (0.06 ng/mL), blood glucose (7 mmol/L), cancer antigen 125 (35.21 U/mL), neuron specific enolase (20.56 ng/mL) and D-dimer (1.41 μg/mL). While the percent of lymphocytes (19%), red blood cell (3.47 × 109/L), hemoglobin (106 g/L), serum potassium (3.32 mmol/L), and albumin (27.1 g/L) were reduced. Other indicators, including GM test, T-SPOT.TB, Xpert MTB (Sputum) and antistreptococcal “o” quantification were negative.

Coughed up sputum smear examination: no bacteria and fungi were found in the microscopic examination, and acid-fast positive bacilli (+) were found in the microscopic examination. It was necessary to further distinguish *Mycobacterium tuberculosis* and non-tuberculous *Mycobacteria*.

**Figure 1:**
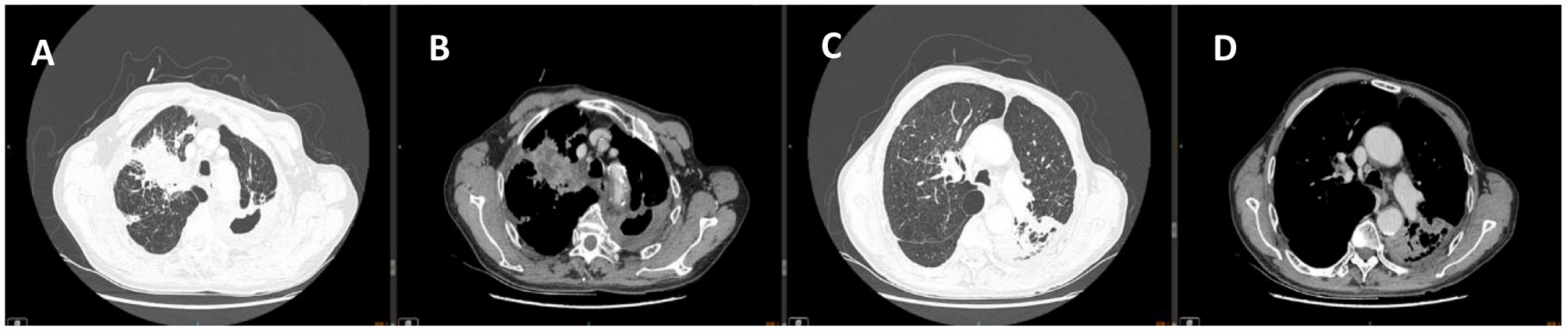
Chest CT of **(A,C)** lung window images showed multiple flaky, strand-like and light mist-like high-density shadows in both lung fields. Chest CT of **(B,D)** corresponding mediastinum window images showed Spot-like calcification, thickened bronchial walls of both lungs, narrowed lumen, and left pleural effusion.

Chest computed tomography scan indicated multiple flaky, strand-like and light mist-like high-density shadows in both lung fields. Visible effusion in the left pleural cavity (Figure 1). Hence, he was preliminarily diagnosed with lung infection and the nature of the upper right lung space to be investigated. We decided to treat the patient with intravenous biapenem for empirical anti-infective and oral rifabutin, ethambutol hydrochloride tablets and clarithromycin sustained-release tablets for anti-tuberculosis treatment, but his condition did not improve.

**Figure 2:**
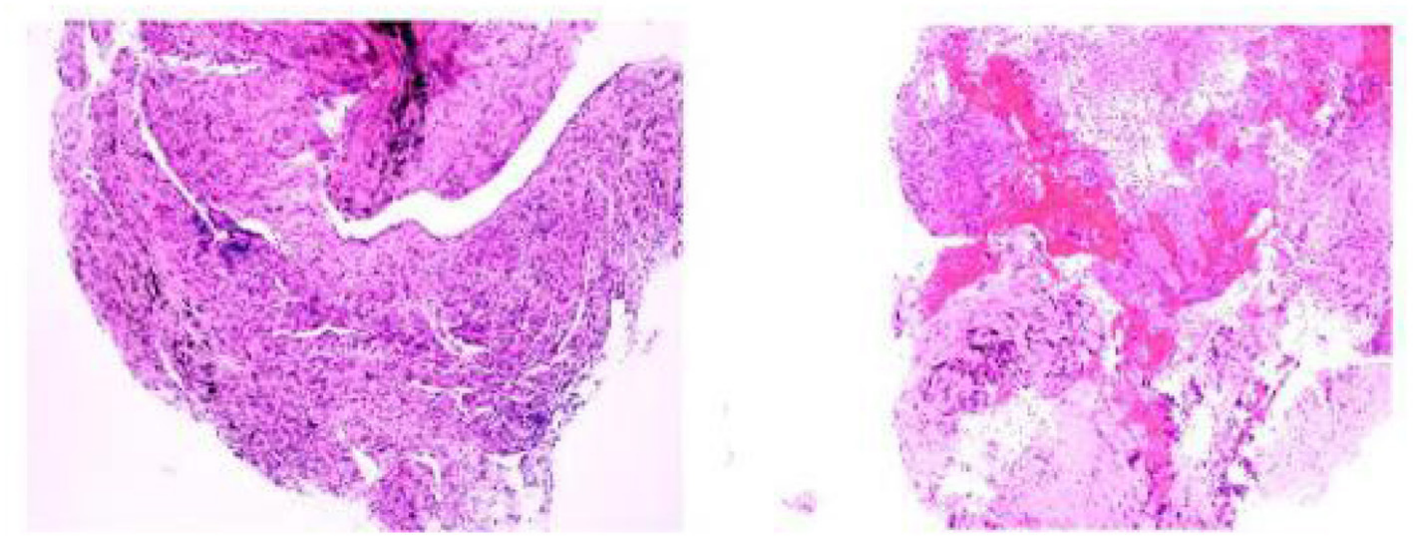
The microscope showed no acid fast bacilli and fungi.

On day 3 of the admission, a bronchoscopy was performed, and the apical bronchus of the right upper lobe was blindly examined. Pathological results showed granulomatous inflammation with necrosis, special staining results showed acid-fast (-), molecular pathological results showed *Mycobacterium tuberculosis* DNA determination (TB-DNA) (PCR) (-) (Figure 2). Bronchoalveolar lavage fluid (BALF) was collected for mNGS and *M. avium complex* was detected (Table 1). Combined with clinical symptoms, laboratory test results and characteristics of MAC, the patient was diagnosed with MAC lung disease. Discontinued biapenem and switched to intravenous amikacin and oral compound sulfa tablets; and continued oral rifabutin, ethambutol hydrochloride tablets and clarithromycin. After 7 days of treatment, the patient's fever, cough, expectoration and wheezing had gradually improved. At the follow-up 3 months later, the patient's above symptoms had been completely relieved.

**Table 1:** Results of mNGS in bronchoalveolar lavage fluid of patient.

**Genus**			**Species**		
**Type**	**Name**	**Sequence number**	**Name**	**Sequence number**	**Relative abundance**
G+	*Mycobacterium*	23	*Mycobacterium avium complex*	2	0.005%

## Discussion

MAC disease is a zoonotic infectious disease caused by MAC infection. According to statistics, MAC is the main strain on all continents of the world (5, 8, 9). In China, the more common infections for NTM-PD are *M. kansasii* and *M. intercelleulare* (10). MAC infection can invade various tissues and organs, including lungs, bone marrow, and lymph nodes. It may also cause diseases of the synovium, bursa, tendon sheath, joint, bone and bone marrow (4, 5). Due to its pathological changes, bacteriological characteristics and antigenic components are similar to *Mycobacterium tuberculosis*, sputum acid-fast staining can be positive. If the bacteria is not further identified, it is easy to be misdiagnosed as *Mycobacterium tuberculosis* infection for a long time, but traditional pathogenic microorganism detection technology has limitations such as low positive rate and long detection cycle, and it is difficult to accurately identify strains, which easily lead to clinical misdiagnosis and seriously affect the prognosis of patients. Especially for patients with positive acid-fast staining of respiratory tract specimens and the anti-tuberculosis treatment is not effective, it is necessary to be highly vigilant against NTM infection. In this case, sputum smear microscopy alone cannot distinguish *Mycobacterium tuberculosis* from NTM. In order to further determine the pathogen, the mNGS detection technology covering the pathogen is used to accurately detect the MAC infection. Combined with the patient's past history of COPD, clinical imaging features and mNGS test results, the final diagnosis was MAC lung disease. This case shows that mNGS has the advantages of high sensitivity and high specificity in the diagnosis and treatment of NTM-PD.

The diagnosis of NTM-PD requires the combination of respiratory system and systemic clinical symptoms, lung imaging findings, and NTM isolation and culture. NTM isolation and culture are the key to diagnosing NTM-PD and distinguishing tuberculosis (11). Based on mNGS does not rely on traditional microbial culture, no specific primers are required, and all DNA/RNA genome information in the sample can be determined in a single run. FEDRIZZI (12) used mNGS to sequence 47 types of NTM strains, including 11 rapidly-growing Mycobacterium strains and 36 slow-growing Mycobacterium strains. At the same time, 41 previously undescribed NTM species were reconstructed and analyzed and expanded the understanding of NTM. Therefore, mNGS has played a huge role in the identification of NTM-PD bacteria, genotyping, clinical diagnosis and treatment, and improvement of patient prognosis.

In the past, due to insufficient knowledge of non-tuberculous mycobacterial infections and limited diagnostic techniques, patients were misdiagnosed as tuberculosis infections, resulting in poor treatment effects and severely affecting the prognosis of patients. In this case, the patient's condition had been repeated for more than 40 years, and eventually progressed to NTM-PD, due to the inability to effectively control the NTM infection. For different NTM infections, the type of medication and the course of treatment are also different (13, 14). Compared with pulmonary infections caused by *Mycobacterium tuberculosis*, NTM-PD has a longer treatment time and a more complicated drug treatment plan. After NTM is tested and identified by mNGS technology, individualized drug treatment can be carried out. Therefore, mNGS detection technology can quickly and accurately detect the types of pathogenic bacteria infected by patients, so as to provide help for clinicians to diagnose and treat patients.

## Conclusion

mNGS is a new type of pathogenic diagnosis technology, which has shown great potential in the identification of pathogenic pathogens of infectious diseases, and has the highest resolution of strain identification. With the increasing popularity and cost reduction of mNGS, it will play an increasingly important role in the diagnosis of NTM diseases. Finding and curing the source of infection in time and reducing contact with patients with NTM are the best measures to prevent NTM infection. Therefore, it is recommended to use mNGS to help clinicians improve the accuracy of identifying *Mycobacterium tuberculosis* and NTM.

## Data Availability Statement

The original contributions presented in the study are included in the article/Supplementary Material, further inquiries can be directed to the corresponding authors.

## Ethics Statement

Written informed consent was obtained from the individuals for the publication of any potentially identifiable images or data included in this article.

## Author Contributions

HZ, J-HL, and Y-LX collected and analyzed patient data. HZ and MZ wrote the manuscript. L-MZ provided supervision. All authors approved the final version. Written consent for publication was obtained from the patient.

## Conflict of Interest

The authors declare that the research was conducted in the absence of any commercial or financial relationships that could be construed as a potential conflict of interest.

## Publisher's Note

All claims expressed in this article are solely those of the authors and do not necessarily represent those of their affiliated organizations, or those of the publisher, the editors and the reviewers. Any product that may be evaluated in this article, or claim that may be made by its manufacturer, is not guaranteed or endorsed by the publisher.
